# Blocking lncRNA *H19*-miR-19a-Id2 axis attenuates hypoxia/ischemia induced neuronal injury

**DOI:** 10.18632/aging.101999

**Published:** 2019-06-05

**Authors:** Zhipeng Xiao, Yongming Qiu, Yingying Lin, Rogelio Medina, Sophie Zhuang, Jared S. Rosenblum, Jing Cui, Zezhi Li, Xiaohua Zhang, Liemei Guo

**Affiliations:** 1 Department of Neurosurgery, Renji Hospital, School of Medicine, Shanghai Jiaotong University, Shanghai 200127, China; 2 Neuro-Oncology Branch, National Cancer Institute, National Institutes of Health, MD 20892, USA; 3 Department of Neurology, Renji Hospital, School of Medicine, Shanghai Jiaotong University, Shanghai 200127, China

**Keywords:** hypoxia, ischemia, Id2, lncRNA H19, miR-19a

## Abstract

Elevated expression of lncRNA *H19* (*H19*) in the setting of hypoxia has been implicated as a promising therapeutic target for various cancers. However, little is known about the impact and underlying mechanism of *H19* in ischemic brain stroke. This study found that *H19* levels were elevated in the serum of stroke patients, as well as in the ischemic penumbra of rats with middle cerebral artery occlusion/reperfusion (MCAO/R) injury and neuronal cells with oxygen glucose deprivation (OGD). Further, knockdown of *H19* with siRNA alleviated cell apoptosis in OGD neuronal cells, and inhibition of *H19* in MCAO/R rats significantly decreased neurological deficit, brain infarct volume and neuronal apoptosis. Lastly, with gain and loss of function studies, dual luciferase reported assay, RNA immunoprecipitation (RIP) and pull-down experiments, we demonstrated the dual competitive interaction of miR-19a with *H19* and the 3’-UTR of Id2 mRNA, resulting in the identification of the *H19*-miR-19a-Id2 axis. With biological studies, we also revealed that *H19*-miR-19a-Id2 axis modulated hypoxia induced neuronal apoptosis. This study demonstrates that the identified *H19*-miR-19a-Id2 axis plays a critical role in hypoxia induced neuronal apoptosis, and blocking this axis may serve as a novel therapeutic strategy for ischemic brain injury.

## INTRODUCTION

Cerebral ischemic stroke continues to incur high morbidity and mortality rates [1]. Therapeutic protocols have continued to evolve as our understanding of the umbra and salvageable penumbra increases. These advances have been broadly aimed at either 1) resolving the infarct in a timely fashion to limit the continued damage incurred by hypoxic stress and resultant neuronal injury or 2) stimulating neural replacement [2]. To date, the application of molecular understanding of hypoxic neuronal injury has been limited [3, 4]. In previous studies, we have demonstrated that inhibitor of DNA binding/differentiation 2 (Id2) promoted hypoxia/ischemia (H/I) induced neuronal apoptosis [5, 6]. Absence of an Id2 targeted inhibitor, however, has limited its application as a potential therapeutic option to protect brain from ischemic injury. Thus, seeking the upstream regulating molecules of Id2 is of great significance.

Long noncoding RNAs (lncRNAs) are transcripts larger than 200 nucleotides that regulate gene expression at the level of chromatin modification, transcription and post-transcriptional processing [7]. LncRNA *H19* (*H19*), the product of *H19* gene, is maternally expressed and paternally imprinted [8], and has been described as an oncogene in various cancers, including bladder, colorectal, gastric and breast cancers [9–12]. Specifically, in the setting of hypoxia, *H19* expression level is upregulated in response to increased activity of hypoxia inducible factor (HIF) [13, 14]. Given this relationship, manipulation of hypoxia-HIF-*H19* axis has been identified as a promising oncologic target [13, 14]. Recent studies have demonstrated a similar elevated *H19* levels following ischemic stroke, however, the exact role of *H19* in the ischemic brain remains unclear [15, 16].

Recently, Luo et al. reported that upregulated *H19* contributed to bladder cancer cell proliferation by increasing Id2 expression [17]. With bioinformatics analysis, Zhao et al. suggested that *H19* regulated Id2 expression through competitive binding of miR-19a/b in acute myelocytic leukemia cells. However, the exact interactive mechanisms between *H19*, miR-19a/b and Id2 remains to be elucidated [18]. More recently, a new regulation mechanism of miRNA sponging was proposed whereby *H19* was thought to function as competing endogenous RNA (ceRNA), thereby modulating the depression of miRNA targets and imposing an additional level of post-transcriptional regulation [19–21]. Taken together, this rationale provided the framework for our investigation into the regulatory mechanisms between *H19*, miR-19a and Id2. In particular, we sought to investigate the role of the *H19*-miR-19a-Id2 axis in the process of H/I induced neuronal injury.

## RESULTS

### Circulating *H19* level was significantly higher in patients with ischemic stroke, and was positively correlated with NIHSS scores

**Figure 1:**
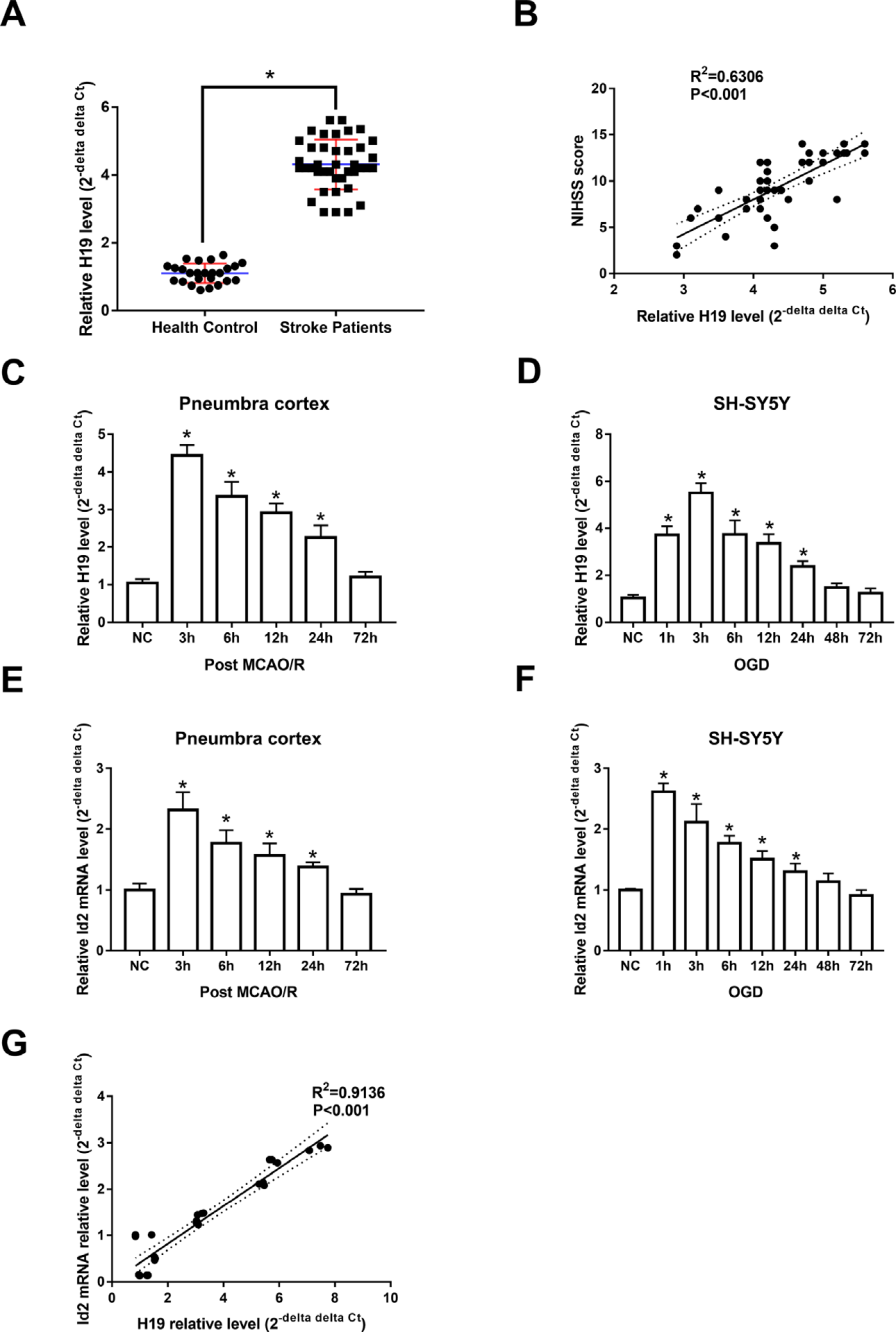
***H19* and Id2 expression levels were significantly elevated in the setting of hypoxia/ischemia**. (**A**) qRT-PCR revealed that *H19* levels in the plasma of ischemic stroke patients were significantly elevated, compared with normal control patients. (**B**) The elevated *H19* levels were positively correlated with patient NIHSS scores within 3 hours of stroke onset. (**C**) In the penumbra cortex of rats, *H19* levels increased and peaked at 3 h, and returned to normal levels at 72 h after MCAO/R. (**D**) In OGD neuronal cells, *H19* levels showed a similar tendency of *in vivo* study. (**E**) qRT-PCR analysis revealed that Id2 mRNA levels in the penumbra cortex of rats increased significantly and peaked at 3 h after MCAO/R in; however, Id2 mRNA levels returned to baseline at 72 h post MCAO/R. (**F**) Similar results were revealed in OGD neuronal cells. (**G**) A correlation analysis revealed that Id2 mRNA level was positively correlated with *H19* level in OGD neuronal cells.

To investigate the involvement of *H19* in ischemic stroke, blood samples from ischemic stroke patients were collected within 3 hours from stroke onset. Quantitative real-time polymerase chain reaction (qRT-PCR) revealed that *H19* was expressed at low level in the plasma of normal control patients, but it was significantly upregulated in ischemic stroke patients (Figure 1A; p<0.05). To further elucidate its clinical significance as a biomarker in ischemic stroke, *H19* levels were assessed in relation to NIHSS scores obtained within 3 hours from stroke onset. Evaluation of these two parameters revealed that *H19* levels were positively correlated with patient NIHSS scores within 3 hours from stroke onset (Figure 1B; p<0.05). These results suggested *H19* could be a diagnostic and prognostic biomarker for ischemic brain stroke.

### *H19* level was also elevated in the penumbra area of MCAO/R rats, as well as in OGD neuronal cells

Animal and cell-based models demonstrated similar trends in *H19* expression levels as those seen in human subjects. Specifically, *H19* levels were significantly elevated in the penumbra cortex of MCAO/R rats and in OGD neuronal cells when compared to controls. *H19* levels significantly increased and peaked at 3 h (p<0.05, respectively), and returned to baseline 72 h after exposure of H/I in both *in vivo* and *in vitro* experiments (Figure 1C and 1D).

### Temporal expression of Id2 mRNA *in vivo and in vitro* models in the setting of H/I, resembling the tendency of *H19* expression

qRT-PCR analysis revealed that Id2 mRNA levels increased significantly and peaked at 3 h after H/I (p<0.05, respectively); however, despite the significant rise, Id2 mRNA levels returned to baseline 72 h after H/I (Figure 1E and 1F). These results suggested that the expression of Id2 mRNA respond to H/I in a specific time dependent manner. Further, since the expression of *H19* and Id2 mRNA demonstrated a similar trend, a correlation analysis was completed and the result revealed that Id2 mRNA level was positively correlated with *H19* level in OGD neuronal cells (R^2^=0.9136, p<0.01) (Figure 1G).

### Inhibition of *H19* decreased Id2 expression and attenuated neuronal apoptosis in OGD neuronal cells

**Figure 2:**
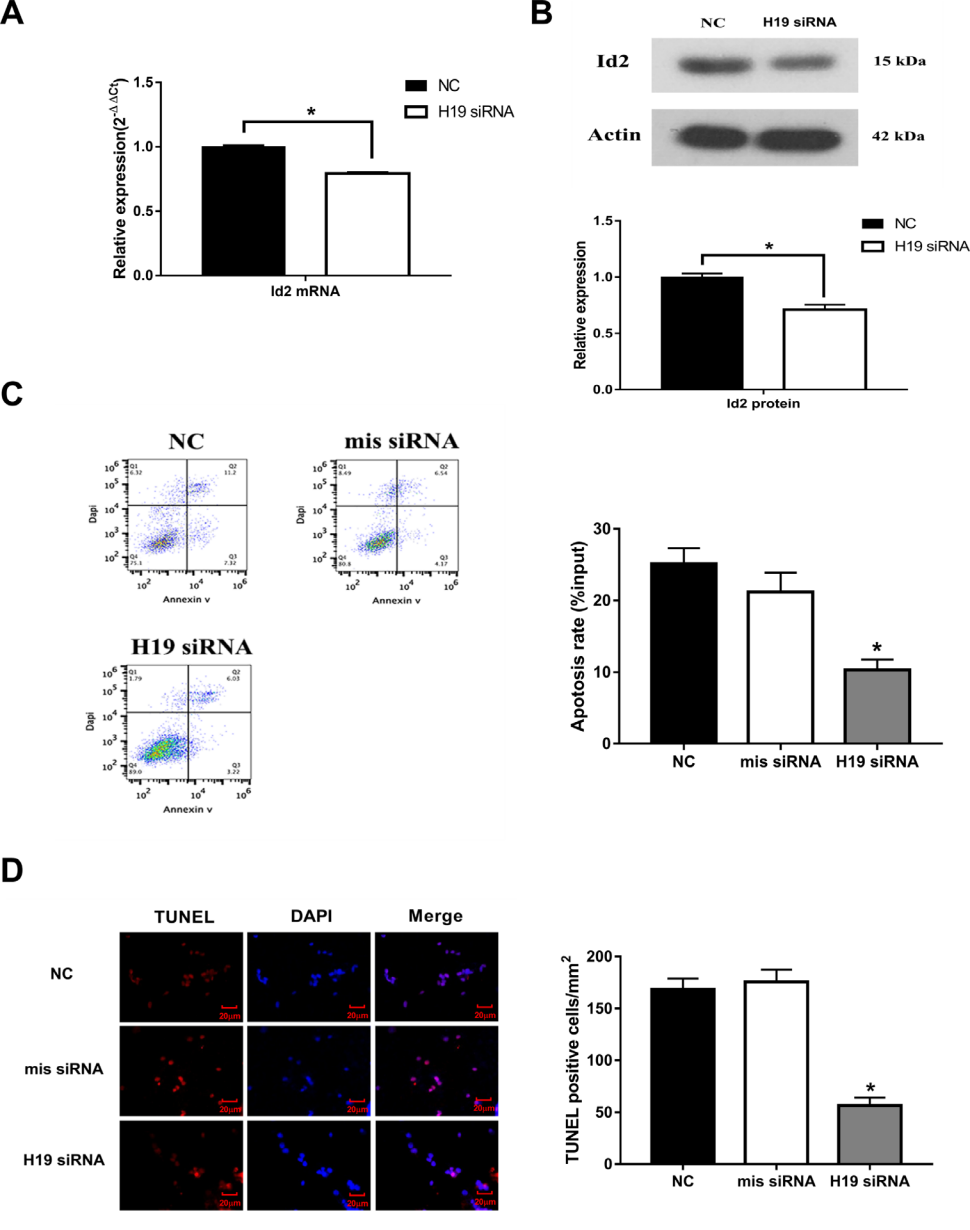
**Knockdown of *H19* decreased Id2 expression, and attenuated neuronal apoptosis *in vitro.*** (**A**) After knockdown of *H19* with siRNA, Id2 mRNA (**A**) and protein (**B**) levels were significantly decreased in OGD neuronal cells. (**C**) Flow cytometric analysis showed a significant decrease in the rate of neuronal apoptosis in OGD neuronal cells of *H19* siRNA group, compared with the NC group or mis siRNA group. (**D**) Immunofluorescence staining demonstrated a similar decrease rate of neuronal apoptosis in *H19* siRNA group.

To further investigate the effect of *H19* on Id2, three different siRNAs were designed against *H19* (named as siRNA-1, siRNA-2, and siRNA-3). All three siRNAs were effective in knocking down *H19* expression (p<0.05, respectively), however, siRNA-1 demonstrated the best efficiency and was therefore utilized in subsequent knockdown experiments (Supplementary Figure 1). With knockdown of *H19*, Id2 mRNA (Figure 2A) and protein (Figure 2B) levels were significantly decreased (p<0.05, respectively). Additionally, flow cytometric analysis and immunofluorescence staining demonstrated a significant decrease in the rate of neuronal apoptosis in the *H19* siRNA group, compared with the NC group or mis siRNA group (Figure 2C and 2D; p<0.01, respectively).

### Inhibition of *H19* attenuated neurological deficits and decreased brain infarct volume in MCAO/R rats

**Figure 3:**
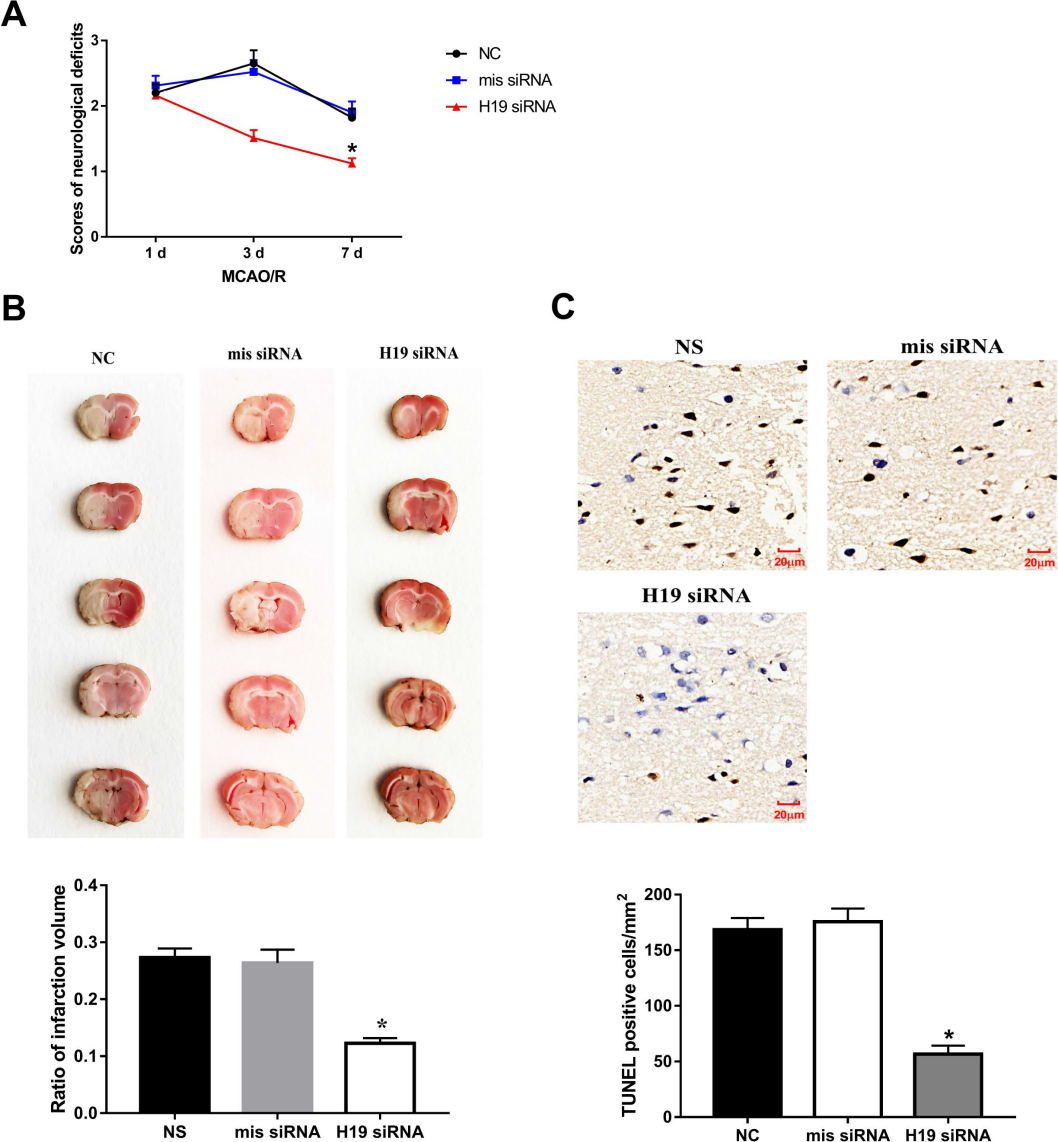
**Knockdown of *H19* reduced neuronal injury and neuronal apoptosis *in vivo.*** (**A**) After a single intracerebroventricular injection of *H19* siRNA in MCAO/R rats, significant reductions in neurological deficit were observed in *H19* siRNA group at 3 d and 7 d post MCAO/R, when compared to the NS group or mis siRNA group. (**B**) Significant reductions in infarct volumes were also seen in rats of *H19* siRNA group, compared with NC group or mis siRNA group. (**C**) There was a significant decrease in the number of TUNEL-positive cells in the *H19* siRNA group compared to NC group or mis siRNA group.

Knockdown of *H19* by a single intraventricular injection of *H19* siRNA offered neuronal protection against ischemic damage following MCA occlusion. Significant reduction in neurological deficit were observed at 3 d and 7 d post MCAO/R when compared to the NS group or mis siRNA group (p<0.01, respectively, Figure 3A). Significant reduction in infarct volumes were also seen in rats injected with *H19* siRNA compared with NC group or mis siRNA group (p<0.01, respectively, Figure 3B). Furthermore, to determine whether the lesser extent of ischemic brain injury from *H19* knockdown was attributable to the reduced neuronal apoptosis, we assessed the frequency of apoptotic neuronal cells in the penumbra area by TUNEL assay at 72 h post MCAO/R. As shown in Figure 3C, there was a significant decrease in the number of TUNEL-positive cells in the *H19* siRNA group compared to NC group or mis siRNA group (p<0.01, respectively).

### *H19* directly interacted with miR-19a

**Figure 4:**
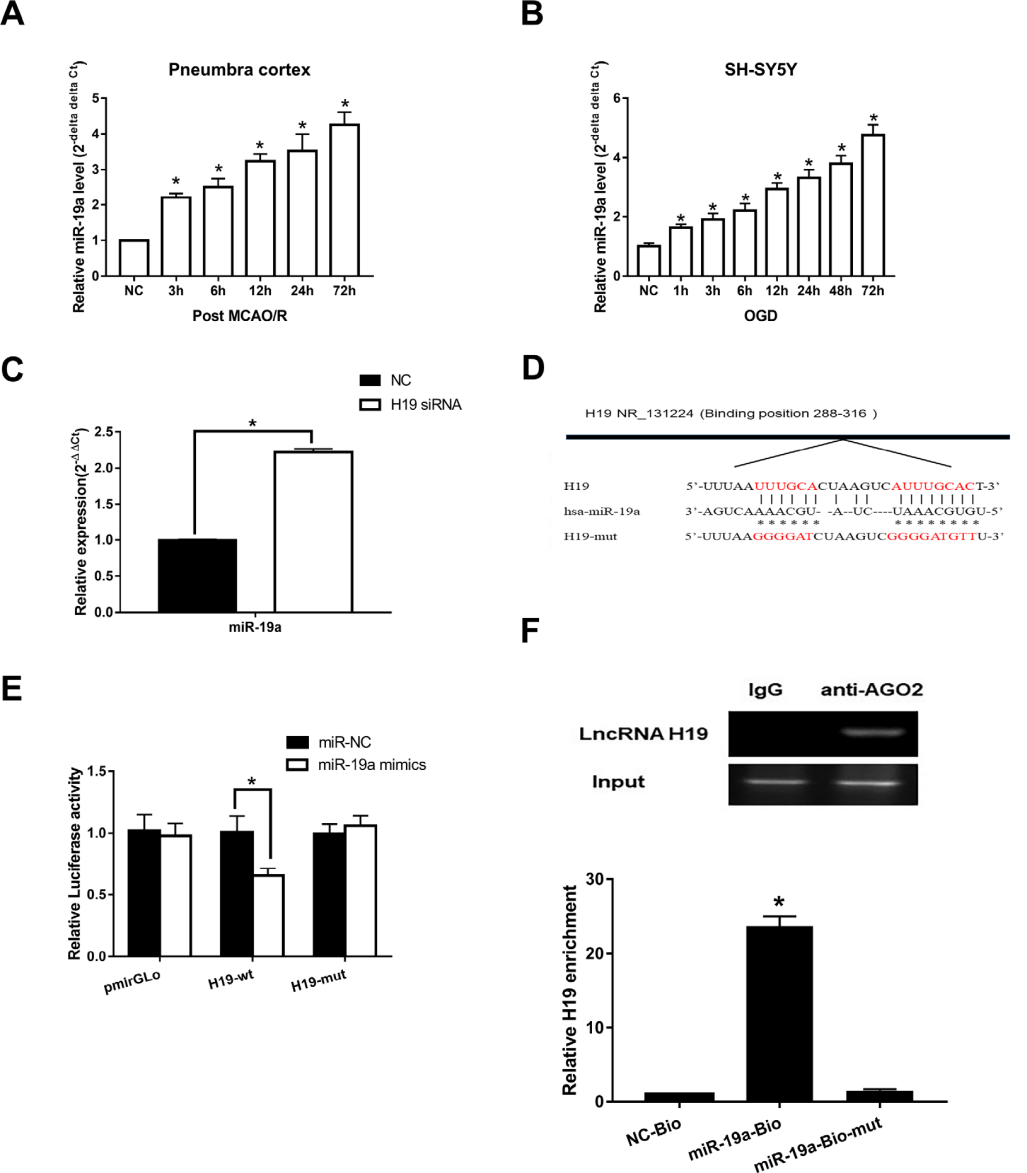
**Direct interaction between *H19* and miR-19a.** (**A**) qRT-PCR analysis revealed that miR-19a levels in penumbra cortex of rats increased gradually and significantly within 72 h after MCAO/R. (**B**) Similar results were observed in OGD models. (**C**) With knockdown of *H19* by siRNA, miR-19a level was significantly elevated in OGD neuronal cells. (**D**) The predicted fragment including miR-19a binding sites on *H19* was cloned into a pmirGLO vector (*H19*-wt), and a mutated vector (*H19*-mut) was also generated by replacing the binding sites with its complimentary sequence. (**E**) Dual-luciferase report assay revealed that miR-19a mimic reduced the luciferase activity of *H19*-wt, but not of *H19*-mut. (**F**) RIP indicated that *H19* was preferentially enriched in Ago2-containing bead compared to those harboring control immunoglobulin G (IgG) antibody. Furthermore, *H19* was pulled down by biotin-labeled miR-19a oligos, but not the mutated oligos.

To assess if *H19* directly interacted with miR-19a, we first determined the levels of miR-19a in the setting of H/I. qRT-PCR analysis revealed that miR-19a levels increased significantly within the first 72 h after H/I in both MCAO/R and OGD models (p<0.05, respectively) (Figure 4A and 4B). Next, we assessed how miR-19a level varied with knockdown of *H19* by siRNA. In the setting of *H19* knockdown, miR-19a levels were significantly elevated in OGD neuronal cells (Figure 4C, p<0.05), suggesting *H19* decreased miR-19a level after H/I.

Further, Starbase 2.0 (http://starbase.sysu.edu.cn) was used to investigate complementary binding patterns between miR-19a and *H19*. Predicted fragment including binding sites (Figure 4D) were cloned into two vectors: a pmirGLO vector (*H19*-wt), and a mutated vector (*H19*-mut) with replacement of binding sites with predicted complimentary sequences. Dual-luciferase report assay revealed that miR-19a mimic reduced the luciferase activity of *H19*-wt, but not of *H19*-mut (Figure 4E, p<0.05). RIP results indicated that *H19* was preferentially enriched in Ago2-containing beads compared to those harboring control immunoglobulin G (IgG) antibody (Figure 4F). Lastly, we performed pull down experiments using biotin-labeled miR-19a oligos. *H19* was pulled down by biotin-labeled miR-19a oligos, but not the mutated oligos (binding sites were mutated to the complement sequences) (Figure 4F; p<0.05). Taken together, these results suggested that miR-19a directly bound to *H19*.

### Id2 mRNA is a direct target of miR-19a

**Figure 5:**
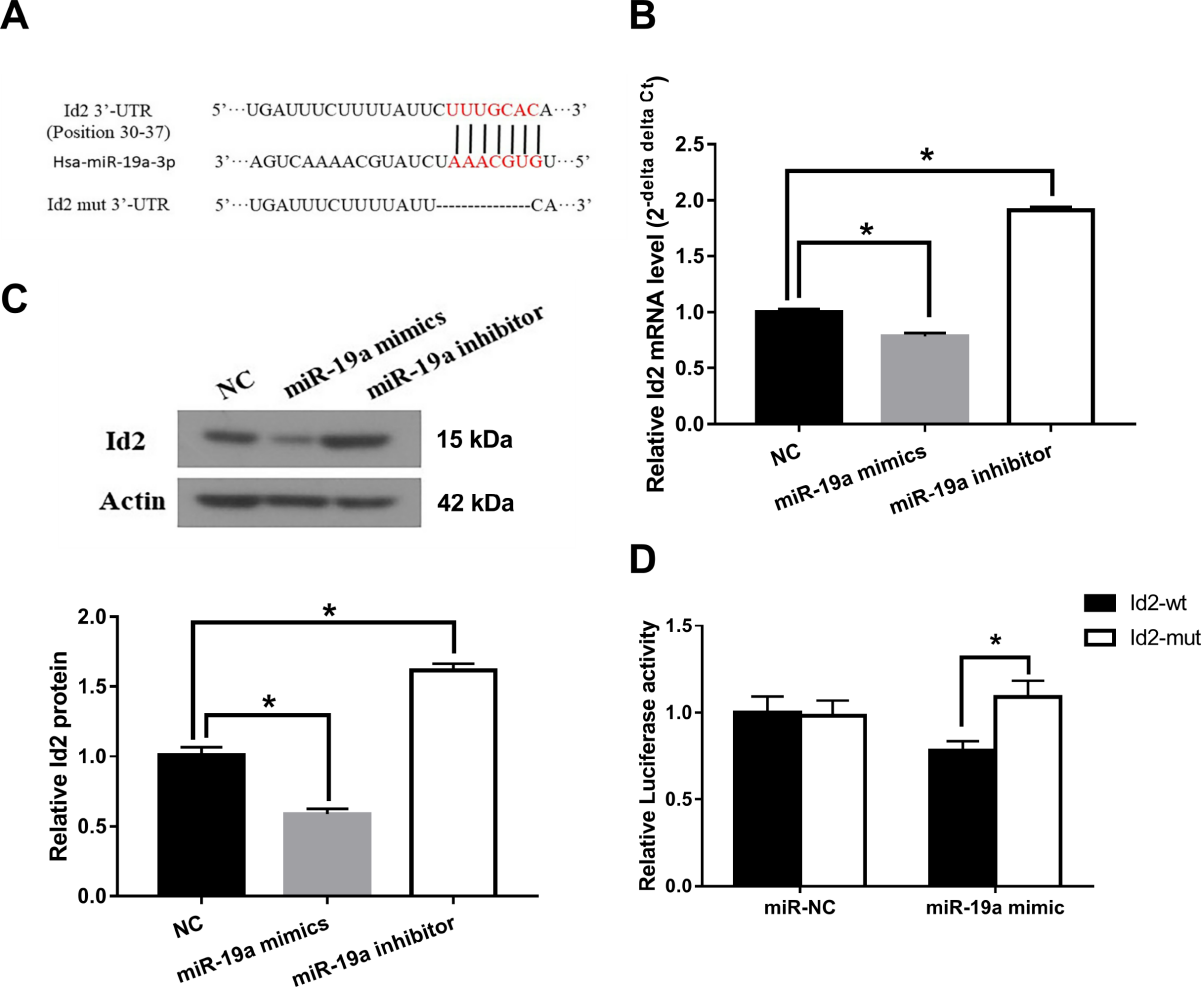
**Id2 mRNA is a direct target of miR-19a**. (**A**) The predicted fragment including binding sites were cloned into the wild-type (Id2-wt) and mutated fragment (Id2-mut). (**B**) miR-19a mimic inhibited the expression of Id2 mRNA in OGD neuronal cells, while miR-19a inhibitor increased its expression. (**C**) miR-19a mimic inhibited the expression of Id2 protein in OGD neuronal cells, while miR-19a inhibitor increased its expression. (**D**) Dual-luciferase report assay demonstrated that miR-19a mimic reduced the luciferase activity of Id2-wt, but not of Id2-mut.

We subsequently investigated the interaction between miR-19a and Id2 mRNA. We used miRanda software (http://www.microrna.org/microrna/home.do) to find miR-19a that could potentially bind to 3′-UTR of Id2 mRNA. The predicted fragment including binding sites were cloned into the wild-type (Id2-wt) and mutated fragment (Id2-mut) (Figure 5A). When treated with miR-19a mimic, the expressions of Id2 mRNA and protein in OGD neuronal cells were inhibited. In contrast, when treated with miR-19a inhibitor, Id2 mRNA and protein expressions increased (Figure 5B and 5C; p<0.05, respectively). Furthermore, dual-luciferase report assay demonstrated that miR-19a mimic reduced the luciferase activity of Id2-wt, but not of Id2-mut (Figure 5D; p<0.05). These results suggested Id2 mRNA is a direct target of miR-19a, which exerted a direct inhibitory effect on Id2 expression via binding 3′-UTR of Id2 mRNA.

### *H19*-miR-19a-Id2 axis modulated hypoxia-induced neuronal apoptosis in vitro

**Figure 6:**
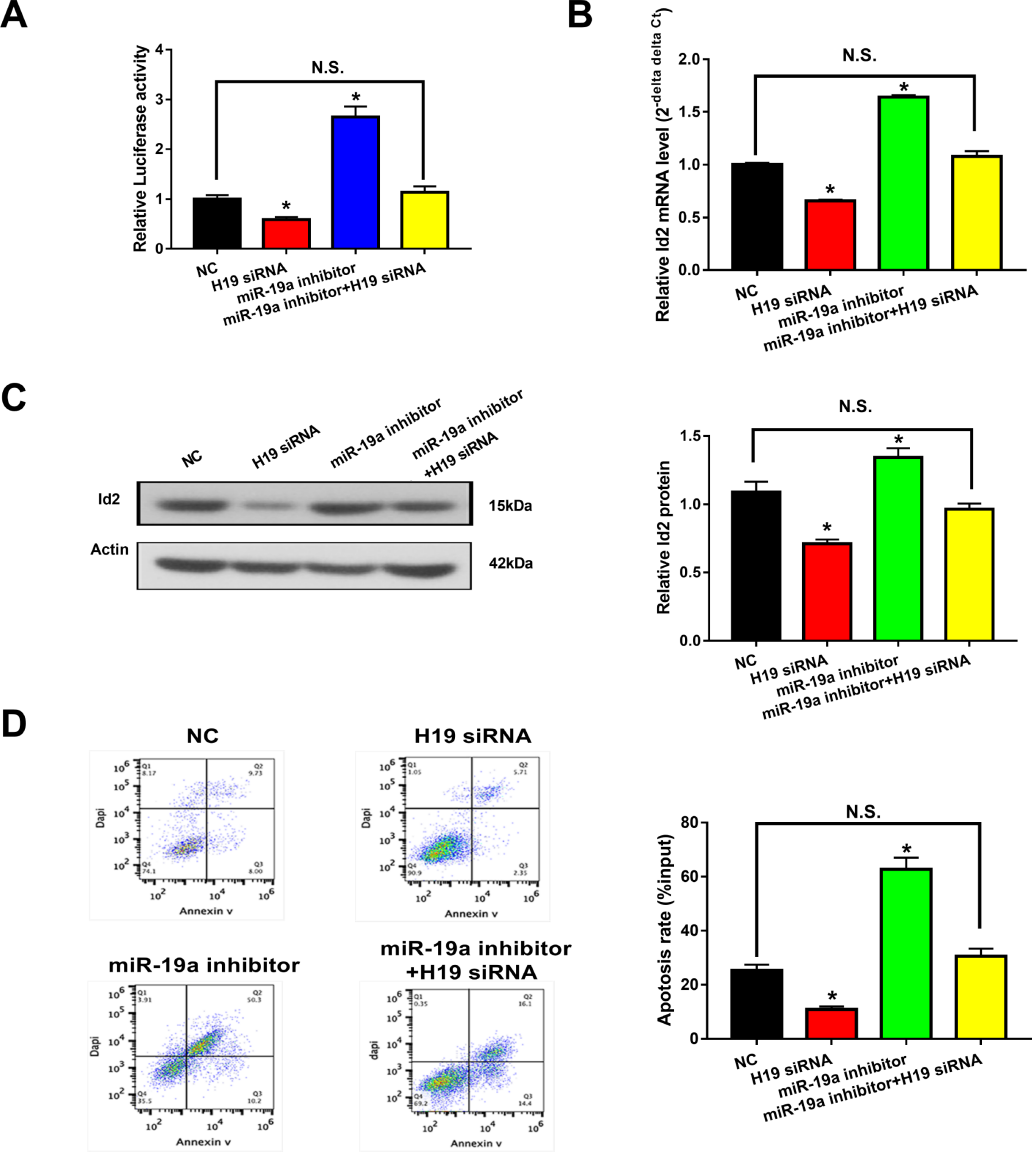
***H19*-miR-19a-Id2 axis modulated hypoxia-induced neuronal apoptosis.** (**A**) Knockdown of *H19* decreased luciferase activity in HEK293T cells transfected with Luc-Id2-3′-UTR; on the other hand, luciferase activity could be rescued by miR-19a inhibitor. In OGD neuronal cells, knockdown of *H19* decreased the expression of Id2 mRNA (**B**) and protein (**C**); decreased expressions of Id2 mRNA and protein levels were attenuated by co-transfection with miR-19a inhibitor. (**D**) Flow cytometric analysis revealed that knockdown of *H19* decreased cell apoptosis in OGD neuronal cells; however, this decrease was relieved by co-transfection of miR-19a inhibitor.

Given the aforementioned results, we hypothesized that the *H19*-miR-19a-Id2 axis modulated hypoxia induced neuronal apoptosis. To test this hypothesis, luciferase reporter plasmids containing the 3′-UTR of Id2 were constructed. As expected, knockdown of *H19* decreased luciferase activity in HEK293T cells transfected with Luc-Id2-3′-UTR (Figure 6A; p<0.05); conversely, luciferase activity was rescued with the introduction of miR-19a inhibitor (Figure 6A; p>0.05). Furthermore, in OGD neuronal cells, knockdown of *H19* decreased the expression of Id2 mRNA and protein (Figure 6B and 6C; p<0.05); while this inhibition was attenuated by co-transfection of miR-19a inhibitor (Figure 6B and 6C; p>0.05). Lastly, flow cytometric analysis revealed that knockdown of *H19* decreased cell apoptosis in OGD neuronal cells (Figure 6D; p<0.05); however, this decrease was relieved by co-transfection of miR-19a inhibitor (Figure 6D; p>0.05). Taken together, these results demonstrated that *H19*-miR-19a-Id2 axis modulated hypoxia induced neuronal apoptosis, and blocking *H19*-miR-19a-Id2 axis could alleviate H/I induced neuronal injury.

## DISCUSSION

In the present study, we first demonstrated that *H19* level was significantly elevated in ischemic stroke patients, *in-vivo* MCAO/R animal model and *in-vitro* OGD neuronal cell model. Further, knockdown of *H19* with siRNA alleviated cell apoptosis in OGD neuronal cells, and inhibition of *H19* in MCAO/R rats significantly attenuated neurological deficit, decreased brain infarct volume and neuronal apoptosis. Lastly, we identified the axis of *H19*-miR-19a-Id2 modulated hypoxia-induced neuronal apoptosis. Taken together, these results suggested blocking of *H19*-miR-19a-Id2 axis may serve as a novel therapeutic strategy for ischemic brain injury.

Generally, *H19* expression was high in embryonic organs and mostly absent or greatly reduced in adult tissues. Of note, *H19* level was demonstrated to be significantly promoted by HIF under hypoxic tumor environments [13, 14]. Similarly, elevated *H19* levels have been implicated in the setting of ischemic stroke [15]. In the present study, we confirmed that *H19* levels were not only significantly elevated in ischemic stroke patients, *in-vivo* MCAO/R rats and *in-vitro* OGD neuronal cells, but also demonstrated circulating *H19* levels were positively correlated with patient NIHSS scores within 3 hours from stroke onset. These results indicated *H19* level could be a diagnostic and prognostic biomarker for ischemic stroke. However, circulating *H19* levels may be a non-specific biomarker, since elevated circulating *H19* levels have been implicated in diverse oncological conditions [9–12, 22–26]. Known oncological etiologies would have to be ruled out prior to designating *H19* level as a more specific biomarker for cerebra ischemia. Perhaps *H19* level in cerebrospinal fluid (CSF) following H/I would render more specific finding. Hence, assessment of *H19* level in the CSF and its specificity in determining neuronal injury in the setting of H/I will be the subject of future studies.

Furthermore, we found that inhibition of *H19* with siRNA reduced neuronal apoptosis *in vitro* and *in vivo.* Recently, Wang et al. reported that *H19* could induce cerebral ischemia reperfusion injury via activation of autophagy, and inhibition of *H19* could protect neuronal cells from OGD induced death [15]; Zhao et al. reported *H19* could induce hippocampal neuronal apoptosis via Wnt signaling, and that inhibition of *H19* might serve as a promising novel target for the treatment of cognitive decline in patient with diabetes mellitus [27]. Therefore, consistent with our study, we suggest that *H19* plays a critical role in H/I induced neuronal injury.

**Figure 7:**
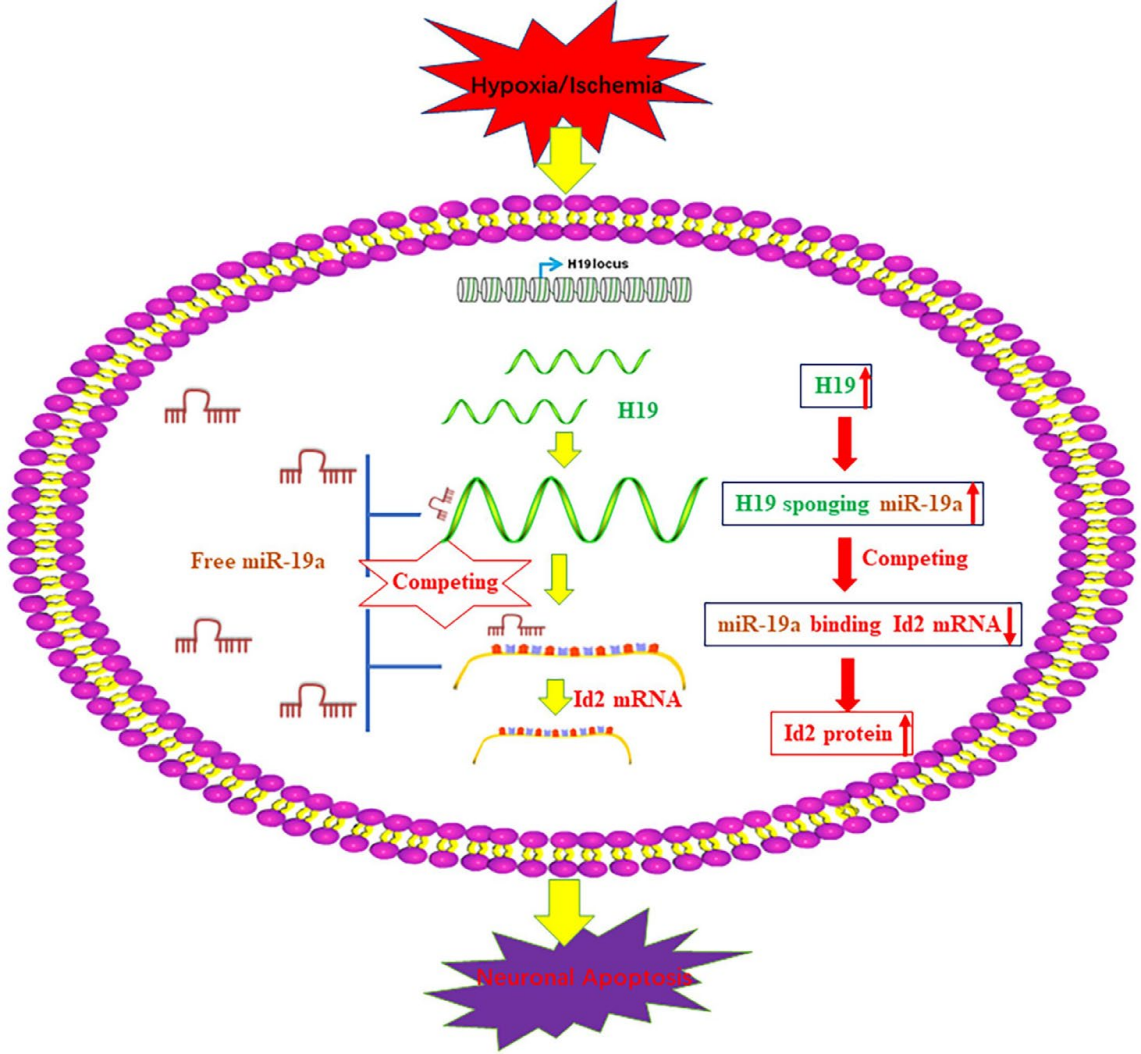
**A schematic figure of the axis of *H19*-miR-19a-Id2.** A schematic figure demonstrates the regulatory mechanism of the *H19*-miR-19a-Id2 axis and its role in modulating H/I-induced neuronal apoptosis.

Interestingly, the elevated *H19* level demonstrated a similar trend as Id2 expression after the exposure of H/I in our *in-vivo* and *in-vitro* studies. Although a similar relationship between *H19* and Id2 has been indicated previously in bladder cancer cells and acute myelocytic leukemia cells, the underlying mechanistic relationship between them have not been well investigated [17, 18]. Inspired by the newly reported regulatory mechanism that *H19* functioned as a competing endogenous RNA (ceRNA) via sponging miRNA [19–21, 28–30], we employed bioinformatics analysis to predict that *H19* promoted Id2 expression via acting as a ceRNA by sponging miR-19a. With dual luciferase reported assay, RIP and pull-down experiments, we demonstrated the dual competitive interaction of miR-19a with *H19* and the 3′-UTR of Id2 mRNA. For the first time, we identified the axis of *H19*-miR-19a-Id2 in the process of H/I induced neuronal apoptosis, and provide a schematic figure outlining the mechanistic relationships of *H19*-miR-19a-Id2 axis (Figure 7). Lastly, we demonstrated that *H19*-miR-19a-Id2 axis modulates H/I-induced neuronal apoptosis and blockage of the axis may have therapeutic benefit in the treatment of ischemic brain injury.

In conclusion, our findings demonstrated *H19* level was elevated in the setting of H/I, and inhibition of *H19* significantly reduced H/I induced neuronal apoptosis *in vivo* and *in vitro*. Further, we also demonstrated that *H19*-miR-19a-Id2 axis played a critical role in hypoxia induced neuronal apoptosis. Thus, blocking of *H19*-miR-19a-Id2 axis could attenuate H/I induced neuronal injury, which sheds new light on the treatment of ischemic brain stroke.

## METHODS

### Study subjects

Forty (40) patients with anterior circulation ischemia and 25 healthy controls were selected from Renji Hospital, School of Medicine, Shanghai Jiaotong University between September 1, 2016, and July 31, 2018. Inclusion criteria were clinical signs and symptoms of focal or global cerebral function loss. Patients who demonstrated new-onset cerebral infarction on magnetic resonance imaging (MRI) within 3 hours from the time of admission were also included in the study. Exclusion criteria included patients with evidence of prior cerebral infarcts and those with the following co-morbidities: diabetes mellitus, coronary artery disease, hypertension, kidney diseases, circulation disorders, or autoimmune diseases. All study subjects underwent routine biochemical tests, cerebral MRIs, and baseline neurological dysfunction assessment using the National Institute of Health Stroke Scale (NIHSS). All study subjects were matched for age (cases, 67.2±9.3; controls, 65.7±10.3) and sex (cases, 25 males to 15 females; controls, 16 males to 9 females). All enrolled patients provided written informed consent, and the study protocol was approved by ethic Committee of Renji Hospital, School of Medicine, Shanghai Jiaotong University.

### Cell culture, and OGD model

SH-SY5Y cells (ATCC) were incubated in Dulbecco’s modified Eagles’s medium (DMEM; Hyclone, USA) supplemented with 10% fetal bovine serum (FBS; Gibco, USA) at 37°C in a humidified atmosphere with 5% CO_2_. HEK-293T cells were cultured in RPMI-1640 medium (Gibco, Grand Island, NY, USA) supplied with 10% fetal bovine serum, 100U/ml penicillin, and 100 μg/ml streptomycin, at 37°C in a 5%CO_2_ atmosphere.

For OGD model, the SH-SY5Y cells were incubated in serum and sugar-free artificial cerebrospinal fluid at 37°C in an atmosphere of 1% O_2_, 94% N_2_ and 5% CO_2_ for 4 h and then incubated in DMEM medium in normoxic atmosphere.

In addition, miR-19a mimics, miR-19a inhibitor and their scramble controls were obtained from Guangzhou RiboBio Co., Ltd. (Guangzhou, China). The siRNAs (named as siRNA-1, siRNA-2 and siRNA-3) directed against *H19* and negative control *H19* scramble were purchased from Invitrogen Life Technologies (Waltham, MA, USA). The sequences of siRNAs were listed in Table 1.

**Table 1:** Sequences of primers and siRNAs used in this study.

**Names**	**Sequences**
H19 siRNA	
siRNA-1	5′-GCAAGAAGCGGGTCTGTTTCT-3′
siRNA-2	5′-GACAAGCAGGACATGACATGG-3′
siRNA-3	5′-GCACTACCTGACTCAGGAATC-3′
mis siRNA	5′-TTGCACGCTAACTTAGGTTCGA-3′
qRT-PCR primers	
H19-F (for human samples and SH-SY5Y cells)	5′-GAAGGCCAAGACGCCAGG-3′
H19-R (for human samples and SH-SY5Y cells)	5′-TCCTCTGTCCTCGCCGTCAC-3′
H19-F (for rat samples)	5′-TTCAAAGCCTCCACGACTCT-3′
H19-R (for rat samples)	5′-GCTCACACTCACGCACACTC-3′
β-actin-F	5′-GTGGCCGAGGACTTTGATTG-3′
β-actin-R	5′-CCTGTAACAACGCATCTCATATT -3′
Id2-F	5′-GGACAGAACCAAACGTCCAG-3′
Id2-R	5′-TAAGCTCAGAAGGGAATTCAGAC-3′
Plasmid construction	
Id2-3′UTR reporter-F	CCGCTCGAGATAAGCGGTGTTCATGAT
Id2-3′UTR reporter-R	ATAAGAATGCGGCCGCTTTGACTTCAGACATGTTT

### Transfection

SH-SY5Ycells were transiently transfected with *H19* siRNAs using Lipofectamine 2000 (Invitrogen, Carlsbad, CA, USA). Briefly, after cultured in normal culture medium for 24 h, the culture medium was replaced by normal culture medium plus Lipofectamine 2000 complex, and cells were incubated in a humidified incubator for 24 h.

### Intraventricular injection of *H19* siRNA

Sprague-Dawley rats weighing 250-300g were obtained from the Experimental Animal Center of Renji Hospital, School of Medicine, Shanghai Jiaotong University. The animal study was approved by the institutional Ethics Committee for Animal Experimentation and conducted in compliance with the Guidelines for Animal Experimentation of this institution. Twenty-four (24) rats were randomly divided into the following 3 groups (n=8 per group): control (intraventricular injection with 0.9% saline), mis siRNA (intraventricular injection with mis siRNA), and *H19* siRNA (intraventricular injection *H19* siRNA). Intraventricular injections were achieved using the following method : 1) rats were anesthetized with intraperitoneal (i.p) injections of pentobarbital (50mg/kg); rats were then placed in stereotactic frame; 2) a 28-guage stainless steel injection cannula was introduced into the right lateral ventricle to deliver single injection according to group (injections were delivered at a rate of 0.5μl/min using a micro-infusion pump and a 10 μl microsyringe); 3) intraventricular cannulas were withdrawn after 5 mins from infusion time at a rate of 1mm/min.

### Rat model of middle cerebral artery occlusion/reperfusion (MCAO/R)

One day after the intraventricular injection of *H19* siRNA, all the rats were performed with the operation of MCAO/R (as previously reported [6]). Briefly, a 3-0 monofilament nylon suture (Beijing Shadong Industrial Corp., China) was introduced into the right internal carotid artery via the external carotid artery until a slight resistance was encountered. The suture was left in place for 90 min and removed to facilitate reperfusion.

### Assessment of neurological deficits for MCAO/R rats

A standardized 0-4 scales was used to evaluate the neurological behavior at 1 day, 3 day and 7 day following MCAO/R [31, 32]. The neurological deficit testing was performed by two observers who were individually blinded to the assignments of the three animal groups.

### Infarct volume measurement

72 h after MCAO/R, the rat brain tissue were collected and the frontal end was sliced and immersed in 2% solution of 2,3,7,-triphenyltetrazolium chloride (TTC) (Sigma Inc., MO, USA) for 30 min at 37°C. Since an infarct tissue does not stain owing to the loss of mitochondrial enzyme activity and a normal tissue will be stained red by TTC, the infarction area that appears white and hemisphere area of each section could be distinguished and quantified by an image analysis system (OlympusImaging ProPlus, Silver Spring, MD, USA). The infarct volume was calculated by dividing infarct area of the ipsilateral hemisphere by total hemisphere.

### Cortex tissue collection and sectioning

The rats were anesthetized by i.p. injection of sodium pentobarbital at 100mg/kg. For RNA and protein expression, the rats were perfused transcardially with 4°C normal saline, and the brain regions that corresponded to the ischemic core and penumbra were dissected on ice according to a previous method [6]. For TUNEL staining, the rats were perfused transcardially with 4% paraformaldehyde (PFA, pH7.4) following 4°C normal saline. Upon removal, the brains were post-fixed in 4% PFA and immediately cryoprotected in 20% and 30% sucrose solution sequentially. A series of coronal sections (25 μm) were made between -1.80 and -4.80 mm bregma levels. Every fifth section totaling 24 sections was used for the staining.

### TUNEL assay

Apoptosis was evaluated on the sections of the cerebral cortices of penumbra area mounted on slides using the Roche TUNEL staining kit (Penzberg, Germany) following the manufacturer’s instructions. The TUNEL-positive cells were counted in five randomized areas per rat brain and expressed as the number of positively stained cells per square millimeter.

### Immunofluorescence

The cells were fixed in 4% formaldehyde for 10 min and were permeabilized in 0.5% Trition X-100 for 10 min and blocked in 1% BSA solution for 45 min. The cells were incubated with the Roche TUNEL staining kit (Penzberg, Germany) following the manufacturer’s instructions. The cells were then counterstained with DAPI (5g/L) for 5 min and observed under fluorescence microscope (Olympus, Japan).

### Flow cytometric analysis

Negative control cells or cells transfected with the desired plasmid were plated in six-well plates. After 48 h incubation, the cultures were incubated with propidium iodide for 30 min in the dark. The percentage of cells in G0/G1, S and G2/M phases of the cell cycle were measured using a flow cytometer (FACS Calibur, BD Biosciences, San Jose, CA, USA) after propidium iodide staining. Cultures were also analyzed for cell apoptosis after double staining with FITC-Annexin V and Propidium iodide (PI). The cells were analyzed with a flow cytometer (FACScan, BD Biosciences) equipped with CellQuest software (BD Biosciences).

### Dual-luciferase reporter assay

Human HEK293T cells were co-transfected with 150 ng of either empty, pmirGLO-NC, pmirGLO-*H19*-wt or pmirGLO-*H19*-mut (Sangon biotech, China). PmirGLO-Id2-wt (Sangon biotech, China) were transfected into HEK293T cells by Lipofectamine 2000 (Invitrogen, USA) -mediated gene transfer, and miR-19a mimics or inhibitor or mis siRNA (NC) were also transfected into HEK293T cells by using the same method. The relative luciferase activity was normalized to Renilla luciferase activity 48 h after transfection. Transfection was repeated in triplicate.

### RNA immunoprecipitation (RIP)

RIP assay was performed using the EZ-Magna RIP^TM^ RNA-Binding Protein Immunoprecipitation Kit (Millipore, MA, USA) according to the manufacturer’s instructions. SH-SY5Y cells at 80-90% confluency were scraped off and lysed in complete RIP lysis buffer. Then, 100 μl of whole cell extract were incubated with RIP buffer containing magnetic beads conjugated with human anti-Ago2 antibody (Cell Signaling, USA), negative control normal mouse IgG (Millipore) and positive control SNRNP70 (Millipore). The co-precipitated RNAs were detected by reverse transcription PCR. Total RNAs (input controls) and IgG were assayed simultaneously to test whether the detected signals resulted from RNAs specifically binding to Ago2.

### Pulldown assay with biotinylated miRNA

SH-SY5Y cells were transfected with Biotinylated miR-19a or biotinylated mutant miR-19a or biotinylated NC (synthesized by Shanghai Gene Pharma Co. Ltd) using Lipofectamine 2000 according to the manufacturer’s instruction. The final concentration of each biotinylated miRNA was 20 nM. The cell lysates were collected 48 h after transfection and incubated with M-280 streptaviden magnetic beads (Invitrogen, San Diego, CA, USA) as described previously [33]. The bound RNAs were purified using TRIzol reagent (TAKALA) for further qRT-PCR analysis.

### Quantitative real-time polymerase chain reaction (qRT-PCR)

To assess *H19* levels in ischemic stroke patients, peripheral blood samples were drawn from these stroke patients within 3 hours of stroke onset, as well as the healthy controls. All blood samples underwent qRT-PCR to measure *H19* levels in serum.

Total RNA in the ischemic patients’ blood serum or the cerebral cortices of penumbra area in MCAO/R rats or OGD SH-SY5Y cells were extracted using TRIzol reagent (Invitrogen, Carlsbad, CA) and further purified with a RNA Micro Kit (Qiagen, Valencia, CA, USA). The following PCR reaction conditions were used when performed in a lightcycler PCR detection system (Roche Diagnostics Ltd., Shanghai, China): 10 min at 95°C, 40 cycles of 10 s at 95°C, 15 s at 60°C, and 20 s at 72°C. Primers for amplification of *H19*, Id2, and β-actin were synthesized by Invitrogen Life Technologies, and are listed in Table 1. The β-actin gene was used as a reference gene, and relative mRNA expression levels were calculated using the standard 2^−ΔΔCt^ method.

### Western blot analysis

Proteins were extracted from the cerebral cortices of the penumbra area in MCAO/R rats or OGD SH-SY5Y cells with RIPA lysis buffer (KenGen Biotech, Nanjing, China) and were quantified using a BCA protein Assay kit (Thermo Fisher Scientific, Inc., Waltham, MA, USA). A total of 30 mg protein lysates were loaded to SDS-PAGE gel, and transferred to PVDF membrane (Millipore, Billerica, MA, USA). The transferred membrane was probed overnight at 4°C with specific primary antibodies (anti-Id2, 1:200 dilution, Santa Cruz Biotechnology; anti-Bax, 1:1000 dilution, Cell signaling technology; anti-β-actin, 1:50000 dilution, Sigma-Aldrich, St.Louis, MO) followed by horseradish peroxidase (HRP)-conjugated goat anti-rabbit secondary antibodies (1:2500 dilution, Promega, Madison, WI, USA). β-actin was used as a control (1:1000; Cell Signaling Technology, Inc., Danvers, MA, USA).

### Statistical analysis

Data was presented as means ± standard deviation and the difference as analyzed by one-way ANOVA followed by Bonferroni’s *post hoc* test. Pearson’s correlation coefficient was used to analyze the correlation between *H19* levels and NIHSS scores, *H19* and miR-19a. A *P*-value less than 0.05 was considered statistically significant.

### Ethics approval

The study was conducted under the approval of the Ethic Committee of Renji Hospital, School of Medicine, Shanghai Jiaotong University. All enrolled patients provided written informed consent. All animal experiments in this study conformed to the principles of the management and use of local experimental animals and followed the Guide for the Care and Use of Laboratory Animals published by the National Institute of Health.

## Supplementary Material

Supplementary Figure
