# Supplementary material for: Blocking lncRNA H19-miR-19a-Id2 axis attenuates hypoxia/ischemia induced neuronal injury
Source: Aging (Albany NY). 2019 Jun 5;11(11):3585–600. doi: 10.18632/aging.101999 (PMC6594804; doi:10.18632/aging.101999)
Supplement: Supplementary Figure [file aging-11-101999-s001.pdf]

SUPPLEMENTARY FIGURE

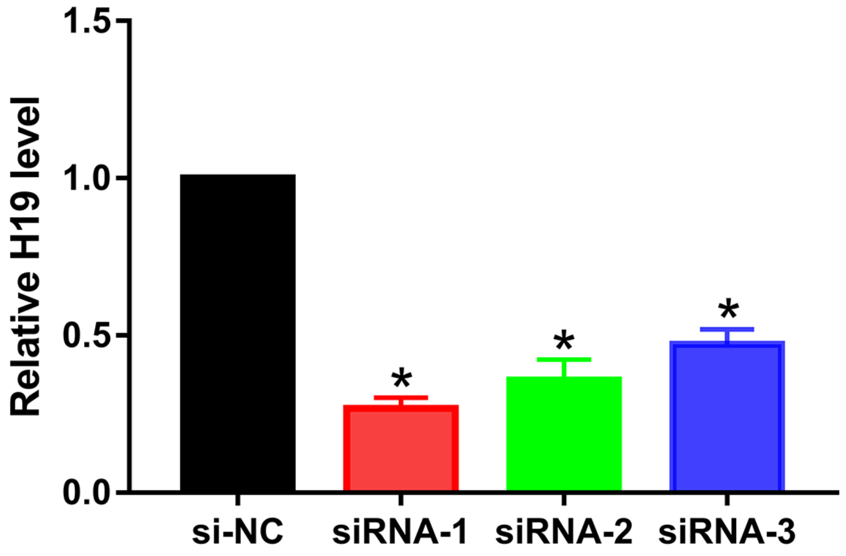

Supplementary Figure 1. The knockdown efficiencies of *H19* siRNAs. All three *H19* siRNAs (named as siRNA-1, siRNA-2, and siRNA-3) were effective in knocking down *H19* expression, however, siRNA-1 demonstrated the best efficiency and was therefore utilized in the subsequent knockdown experiments.
